# Assessment of implementation methods in sepsis: study protocol for a cluster-randomized hybrid type 2 trial

**DOI:** 10.1186/s13063-023-07644-y

**Published:** 2023-09-29

**Authors:** Hannah E. Frank, Laura Evans, Gary Phillips, RPhillip Dellinger, Jessyca Goldstein, Lori Harmon, David Portelli, Nima Sarani, Christa Schorr, Kathleen M. Terry, Sean R. Townsend, Mitchell M. Levy

**Affiliations:** 1 Department of Psychiatry and Human Behavior, The Warren Alpert Medical School of Brown University, Providence, RI USA; 2 Division of Pulmonary, Critical Care and Sleep Medicine, University of Washington, Seattle, WA USA; 3 Biostatistical Consultant, Center for Biostatistics, The Ohio State University, Retired From, Columbus, OH USA; 4 Critical Care Division, Cooper University Health Care, Cooper Medical School of Rowan University, Camden, NJ USA; 5 Division of Pulmonary, Critical Care and Sleep Medicine, The Warren Alpert Medical School of Brown University, Providence, RI USA; 6 Society of Critical Care Medicine, Mount Prospect, IL USA; 7 Department of Emergency Medicine, The Warren Alpert Medical School of Brown University, Providence, RI USA; 8 Department of Emergency Medicine, University of Kansas Health System, Kansas City, KS USA; 9 Cooper Research Institute, Cooper University Health Care, Cooper Medical School of Rowan University, Camden, NJ USA; 10 Lightning Strategies LLC (WOSB), New York, NY USA; 11 California Pacific Medical Center, San Francisco, CA USA

**Keywords:** Sepsis, Septic shock, Sepsis bundles, Implementation science

## Abstract

**Background:**

Sepsis is the leading cause of intensive care unit (ICU) admission and ICU death. In recognition of the burden of sepsis, the Surviving Sepsis Campaign (SSC) and the Institute for Healthcare Improvement developed sepsis “bundles” (goals to accomplish over a specific time period) to facilitate SSC guideline implementation in clinical practice. Using the SSC 3-h bundle as a base, the Centers for Medicare and Medicaid Services developed a 3-h sepsis bundle that has become the national standard for early management of sepsis. Emerging observational data, from an analysis conducted for the AIMS grant application, suggest there may be additional mortality benefit from even earlier implementation of the 3-h bundle, i.e., the 1-h bundle.

**Method:**

The primary aims of this randomized controlled trial are to: (1) examine the effect on clinical outcomes of Emergency Department initiation of the elements of the 3-h bundle within the traditional 3 h versus initiating within 1 h of sepsis recognition and (2) examine the extent to which a rigorous implementation strategy will improve implementation and compliance with both the 1-h bundle and the 3-h bundle. This study will be entirely conducted in the Emergency Department at 18 sites. A secondary aim is to identify clinical sepsis phenotypes and their impact on treatment outcomes.

**Discussion:**

This cluster-randomized trial, employing implementation science methodology, is timely and important to the field. The hybrid effectiveness-implementation design is likely to have an impact on clinical practice in sepsis management by providing a rigorous evaluation of the 1- and 3-h bundles.

**Funding:**

NHLBI R01HL162954.

**Trial registration:**

ClinicalTrials.gov NCT05491941. Registered on August 8, 2022.

**Supplementary Information:**

The online version contains supplementary material available at 10.1186/s13063-023-07644-y.

## Background

Sepsis is the leading cause of admission to intensive care units (ICUs), the leading cause of death in ICUs, and the most common cause of hospital readmissions [1–6]. Sepsis is also the most expensive condition treated in the USA [7]. The Surviving Sepsis Campaign (SSC) was formed in 2002 to reduce sepsis mortality. SSC developed evidence-based guidelines in 2004, and updated guidelines in 2008, 2013, 2017, and 2021 [8–11]. Since simply publishing guidelines seldom leads to changes in clinical behavior [12], the SSC in partnership with the Institute for Healthcare Improvement [13] developed sepsis “bundles” to change clinical practice. A bundle aggregates evidence-based practices into a discrete management approach. Initial 6- and 24-h SSC bundles were revised to 3- and 6-h bundles, and subsequently to the 1-h bundle [14].

Since the advent of sepsis bundles, multiple observational studies have demonstrated an association between implementation of sepsis bundles and improved survival [15–35]. Adoption has been widespread [16, 36–38]. Higher bundle compliance is associated with lower mortality [39–41]. Based on these data and since sepsis is a “medical emergency” [7], all 3-h bundle elements were included in the 1-h bundle in recognition that earlier management might improve survival [14]. The 1-h bundle was named to differentiate the importance of initiating all the elements within that hour as opposed to completion. Although some hospitals have adopted the 1-h bundle, widespread adoption has not occurred.

In 2015 the Centers for Medicare and Medicaid Services (CMS) introduced “The Early Management Bundle: Severe Sepsis/Septic Shock” (SEP-1), which requires hospitals to report compliance with certain clinical processes related to sepsis care. SEP-1 focuses on timely recognition and early intervention. Despite the CMS reporting requirement, hospital compliance remains moderate at 60% [42].

Although bundle compliance would appear essential to improve sepsis outcomes, implementation science approaches to improve bundle compliance have not been evaluated. In particular, there is no widely accepted implementation strategy (i.e., a method to enhance adoption of identified practices) [43] to promote compliance with sepsis bundles. Application of rigorous implementation science techniques, identifying both barriers and facilitators, may improve bundle compliance [44].

This pragmatic, cluster-randomized hybrid type 2 effectiveness-implementation study will compare the effectiveness of the 1-h bundle to the 3-h bundle. The implementation strategy will be guided by the EPIS (Exploration, Preparation, Implementation, Sustainment) Framework [45], a commonly used approach that delineates phases of implementation and associated constructs. This study will explicitly test rigorous implementation strategies and incorporate formative, process, and summative evaluations throughout. In addition, sepsis phenotypes will be identified to probe how sepsis bundles may interact with a specific clinical presentation.

## Methods

### Study design

**Fig. 1:**
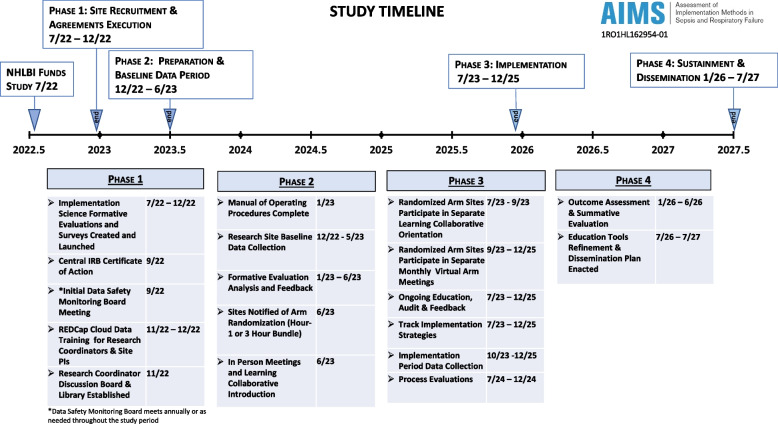
Study timeline

**Fig. 2:**
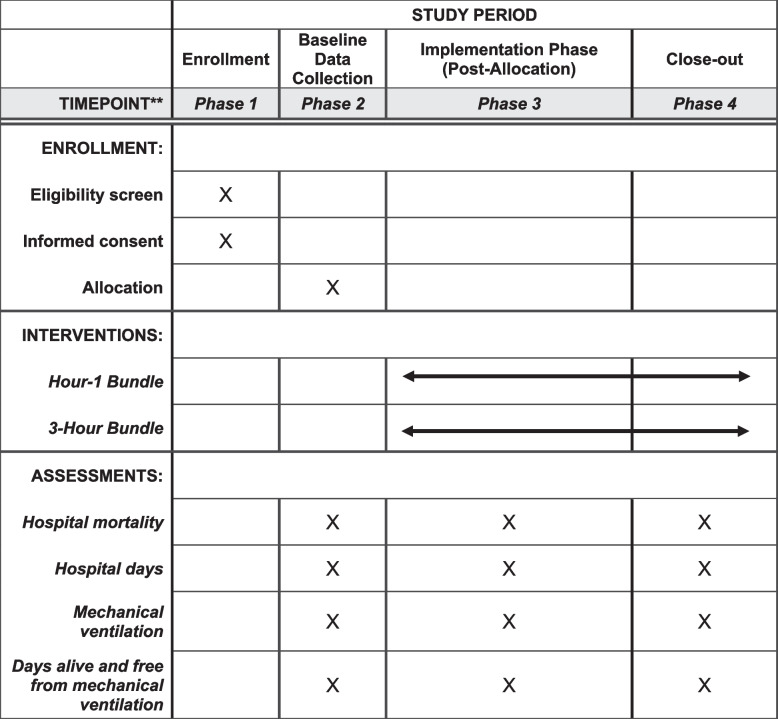
SPIRIT checklist: Schedule of enrollment, interventions and assessment

This study is a pragmatic, cluster-randomized trial of 18 hospitals (9 in each arm) comparing the 1- and 3-h bundles in patients presenting to emergency departments (ED) with sepsis. We will use a hybrid type 2 effectiveness-implementation design [46] to evaluate (1) effectiveness outcomes, including mortality and respiratory failure, and (2) implementation outcomes, including compliance (Fig. 1). The multi-faceted implementation strategy will apply to both trial arms. Application of the Pragmatic Explanatory Continuum Indicator Summary [47–50] (PRECIS) tool identifies this trial as pragmatic (vs. explanatory) [51]. A cluster-randomized trial is appropriate for analyzing the comparative effectiveness of the 1-h bundle versus the 3-h bundle because of the impossibility of randomizing patients to a particular approach and the importance of implementation of bundles at the hospital level. This protocol adheres to the Standard Protocol Items: Recommendations for Interventional Trials (SPIRIT) guidelines [52]. Figure 2 provides a summary of the enrollment schedule, interventions, and assessments using the SPIRIT flow diagram.

### Study objectives

The study aims are as follows: (*Aim 1*) Examine comparative effectiveness of the 1-h versus 3-h sepsis bundle on the primary outcome of hospital mortality and secondary outcomes of length of stay, ventilator-free days at 28 days, and rate of acute respiratory failure requiring mechanical ventilation. *Hypothesis:* The 1-h bundle will have lower hospital mortality. (*Aim 2*): Conduct a mixed methods evaluation of a multi-faceted implementation strategy to enhance compliance with the 1 and the 3-h bundles. *Hypothesis*: 1 and 3-h bundle implementation compliance will be similar as assessed by (a) the electronic health record (EHR), (b) surveys of key informants regarding implementation climate, organizational readiness for change, and implementation leadership, (c) recordings of learning collaborative meetings, and (d) interviews with participants. (*Aim 3*): Determine if clinical sepsis phenotypes determined by characteristics measured at presentation modify the treatment effect of the 1 or 3-h bundle. *Hypothesis:* Routine clinical information available at ED presentation will identify phenotypes more likely to benefit from the 1 or 3-h bundle.

### Study setting

We will conduct this study at 18 diverse acute care hospitals in the USA. Hospital selection was based upon responses of ED leaders to a baseline survey to ensure a diverse and representative sample of hospitals. Considerations included hospital size, teaching status, and known improvement strategies. Discovery, the Society of Critical Care Medicine’s (SCCM) critical care research network, will serve as clinical and data coordinating center for the study. Sites were recruited from Discovery and via polling emergency medicine members of SCCM as to suitability of their hospital as a site. Both academic and community hospitals are included to support the pragmatic nature of the study and ensure generalizability. To account for baseline implementation efforts, prior to randomization, sites that reported less than 30% or more than 70% compliance with SEP-1 were not included for study participation. Patient inclusion criteria include suspected severe sepsis and/or septic shock in the ED with 2 out of 4 Systematic Inflammatory Response Syndrome (SIRS) criteria, evidence of organ dysfunction, and suspected infection—consistent with the criteria established for SEP-1 by CMS. Time zero (the beginning of measuring compliance with the 3-h bundle) is defined in the same manner as described by CMS—all three criteria have to be met within 6 h in order for the definition of time zero to be met. Patients will be excluded if, within 24 h of admission, they are made do-not-resuscitate/do-not-intubate, comfort measures only, or considered not eligible to full aggressive care because of patient or family wishes. For consistency, only hospitals utilizing Epic as their EHR were included. In addition, the decision was made by the investigators that if patients do not present with hypotension, but develop hypotension after the criteria for time zero are met, then no fluids will be given. This does not mean that no fluids will be given to a patient, but rather that, for the purposes of the AIMS study compliance with fluids will not be measured.

### Study committees

In addition to coordinating responsibilities overseen by the project manager (LH) at SCCM, the conduct of the study will be overseen by a steering committee and a Data and Safety Monitoring Board (DSMB). The DSMB is independent of the steering committee and study sponsor. It will meet annually to assess the safety and efficacy of study procedures, monitor the overall conduct of the study, and ensure that data is collected reliably. The steering committee will meet every other week to discuss trial management and will be comprised of the principal investigator (ML), the head of implementation science (HEF), the heads of the subcommittees (CS, DP, NS, and LE), a study consultant with experience from the New York State sepsis initiative (KMT), and the research associate (JG). The subcommittees include (1) the Data Subcommittee (chaired by CS): Charged with building the clinical report form, managing data entry, producing videos to describe data entry procedures, and reviewing missing data; (2) the Education Subcommittee (chaired by DP and NS): Charged with producing educational algorithms for posting in emergency departments and developing educational slide sets for distribution to all sites; and (3) the Publication Subcommittee (chaired by LE): Charged with developing policies for all study publications, including for authorship and manuscript proposals. All subcommittees will report to and receive input from the steering committee.

### Randomization

Hospitals will be randomized to one of two arms (1- or 3-h bundle) in a 1:1 ratio. Randomization will be accomplished using randomly permuted blocks of variable size and controlling for academic or community status. Hospital physician and nurse dyads will be told which condition they were randomized to during in-person meetings at SCCM immediately prior to the Implementation Phase; separate meetings will take place for each study arm. Each arm will undergo a 30-month structured, collaborative implementation approach to operationalize compliance with their bundle assignment. The elements of both bundles are identical—only the timing is different (see Table 1). In this cluster-randomized clinical trial, the hospital is the unit of randomization. Hospitals will be informed as to their randomized arm after the baseline collection period. The randomization will be done by the study statistician and held only in the statistician’s computer until the reveal meeting at the end of the baseline period. Due to the pragmatic, open-label design of this trial and its objective measurement of study outcomes, the study staff, investigators, sites, and biostatisticians will not be masked to treatment condition once randomization occurs. Patients will receive standard of care for sepsis and their data will be collected retrospectively by the hospital each month.

**Table 1:** 1- and 3-h bundle elements: quality and outcome indicator definitions and specifications

	**Definition of bundle element (quality indicator) or outcome measure obtained from the EHR by site staff**	**Both study arms**
**Bundle element**		
1	Indicate if patient with sepsis or septic shock had an initial lactate drawn	Initiated or completed within 1 or 3 h following the time of presentation in the ED
2	Indicate if patient had blood cultures collected prior to broad-spectrum antibiotics for sepsis and/or septic shock	Initiated or completed within 1 or 3 h following the time of presentation in the ED
3	Time in minutes to receipt of broad-spectrum antibiotics for sepsis and/or septic shock following the time of presentation in the ED	Initiated or completed within 1 or 3 h following the time of presentation in the ED
4	Indicate if patient had 30 ml/kg IVF bolus initiated if hypotensive or with a lactate ≥ 4 mmol/L on presentation in the ED	Initiated or completed within 1 or 3 h following the time of presentation in the ED or n/a
5	Indicate if patient with persistent hypotension received vasopressors to achieve mean arterial pressure > 65 mmHg	Initiated or completed within 1 or 3 h following the time of presentation in the or n/a
6	The percent of cases of sepsis and/or septic shock that completed all elements of the bundle	Initiated or completed within 1 or 3 h following the time of presentation in the ED
7	The percent of all patients that completed all elements of the bundle	Initiated or completed within 1 or 3 h following the time of presentation in the ED
**Outcome measures**		
A	Hospital mortality for patients with sepsis and/or septic shock (primary outcome)	Yes vs. no
B	Hospital days for patients with sepsis and/or septic shock	Count
C	Indicate if mechanical ventilation present during admission for patients with sepsis and/or septic shock	Yes vs. no
D	Days alive and free from mechanical ventilation	Count

### Implementation strategies

The implementation package consists of six strategies (see Table 2) from the Expert Recommendations for Implementing Change (ERIC) [53] strategy taxonomy and carried out by the study team according to the Exploration, Preparation, Implementation, Sustainment (EPIS) [54] phase. The only difference in the implementation package for the two arms is training specific to implement the bundle assignment.

**Table 2:** Overview of implementation strategies by study phase

Implementation strategy	EPIS phase
Change the record system	Exploration (6 months)
Implementation formative evaluation: Assess Readiness and barriers and facilitators	Exploration (6 months)
Identity and prepare champions	Adoption/preparation (6 months)
Create a learning collaborative	Adoption/preparation (6 months)
Conduct ongoing trainings	Implementation (30 months)
Develop and implement tools	Implementation (30 months)
Audit and feedback	Implementation (30 months)
Implementation process evaluation	Implementation (15 months)
Implementation summative evaluation	Sustainment (6 months)
Distribute educational materials	Sustainment (6 months)

#### ***Exploration phase (6 months) (***Fig. 1***)***

##### Assess for readiness, organizational culture, and barriers and facilitators

Prior to implementation, sites will participate in formative evaluations [55], which will involve: First, *surveys with ED leadership* (physician and nurse dyads) to explore leadership and team attitudes and engagement, assess their needs related to sepsis bundle adoption, explore anticipated barriers to implementation, and identify potential facilitators of change. We will use several scales during the exploration phase, and then again at the conclusion of the Implementation phase: (a) Implementation Leadership Scale (ILS) [56]; (b) Evidence-Based Practices Attitudes Scale (EBPAS-15) [57]; (c) Organizational Readiness for Implementing Change (ORIC); and (d) Implementation Climate Scale (ICS) [58]. Secondly, *semi-structured interviews with key ED informants* will occur during the formative, process, and summative evaluation phases [59]. Leaders, physicians, nurses, and other hospital staff will be asked about their openness/resistance to change generally and to the use of sepsis bundles, beliefs about the evidence supporting sepsis bundles, perceptions of the utility of the bundles, fiscal obstacles (or facilitators), and beliefs about the importance of timely treatment.

#### Adoption-preparation (baseline data collection) phase (6 months)

Staff at each site will be trained to enter data into SCCM’s research electronic data capture (REDCap) [60, 61]. These data will serve as a baseline for evaluating the change in bundle compliance at the end of the 30-month implementation phase associated with the implementation strategy.

##### Identify and train champions

Sites will choose a nurse and physician as site champions (i.e., clinicians supportive of implementation [39, 62]). Aggregate results from exploration phase surveys will be shared with site investigators at an in-person meeting hosted at SCCM.

##### Create a learning collaborative

Each site will develop a change team consisting of an ED physician, ED nurse, hospital quality improvement advisor, and data collector. A monthly virtual meeting will be conducted by the study team for each arm with the site teams, including review of educational approaches and materials.

#### Implementation phase (30 months)

##### Conduct separate ongoing training virtual meetings for each arm

Following randomization, site teams will participate in virtual monthly learning collaborative meeting, focusing on content of the sepsis bundles, creating a team atmosphere and how to review and overcome barriers to implementation. Each learning session will include additional instruction on facilitating change, developing an information infrastructure to measure progress and quality improvement and implementation science theory. Sites in each arm will have access to a distinct on-line discussion forum to share ideas, questions, challenges, and solutions.

##### Develop and implement tools for quality monitoring (reporting tool)

Sites will (1) provide ongoing feedback and training of data collectors, (2) develop automated EHR alerts for identification of sepsis in the ED, (3) review order sets, and (4) assess physician and nurse engagement.

##### Audit, feedback and process evaluation of bundle implementation

Hospital sites in both arms will receive feedback on compliance every month [62, 63] including (1) review of successes and failures of implementation, (2) ongoing discussion of compliance barriers, and (3) methods for overcoming barriers. Learning collaborative meetings will be recorded for analysis in formative process evaluations.

#### Sustainment phase (12 months)

##### Outcome assessment and summative evaluation (6 months)

At the end of the 30-month implementation period, sites will continue to implement the 1 and 3-h bundles. Data collection and reporting will continue post-intervention to assess sustainability. Data collected during the 6-month outcome assessment phase will serve as the primary data set for analysis and for comparison to the 6-month period of baseline data. A mixed methods summative evaluation to measure change in barriers and facilitators identified in the exploration phase and bundle sustainment will be completed. We will administer the ILS [56], EBPAS-15 [57], ORIC, and ICS [58] measures, as well as the 40-item Program Sustainability Assessment Tool [64]. Semi-structured interviews will be repeated with key ED team members evaluating the EPIS framework and most effective implementation strategies.

##### Dissemination activities (6 months)

Following the data analysis, investigators will develop educational materials that summarize the findings of the trial as well as “how to guides” for generalizing the implementation strategy to other hospitals [65–68]. Investigators will disseminate results to national stakeholders (i.e., SCCM, Institute for Healthcare Improvement, professional societies, and hospital associations). Specifically, as indicated via letters of support, SCCM and the American College of Emergency Physicians (ACEP) will disseminate trial results through their communication channels, including discussion groups and social media accounts. In addition, trial findings will be published in peer-reviewed journals, presented at national and international conferences, and shared by study investigators via social media. Furthermore, de-identified data will be made available in a data repository in adherence with NIH’s Data Sharing policy.

### Recruitment and power

The study sample size will (4070 in each arm) provide adequate power to detect a difference-in-difference in hospital mortality between the pre-intervention period and the post-intervention period. This estimate is based upon the number of patients who receive care compliant with the respective bundles and associated hospital mortality. Analyzing ED patients included in the New York Sepsis Database, pre-intervention hospital mortality was 19.1% for those compliant with the 3-h bundle. Analyzing the data to create cohorts compliant with therapies at 1 h and at 3 h, the post-intervention hospital mortality of those receiving care compliant with the 1-h bundle was 16.2% and hospital mortality for those receiving care compliant with the 3-h bundle was 18.9% (preliminary data submitted to NHLBI R01 HL153268). This is based on the assumption that in the post-intervention period, the median time to antibiotics will be reduced by a quarter of an hour (25% reduction) in the 1-h arm. This quarter of an hour reduction in time to antibiotics translates to a 0.2% reduction in hospital mortality. Table 3 summarizes the assumptions used in the power calculation.

**Table 3:** Assumptions for Donner and Klar’s cluster-randomized power calculation using a mixed model test for two proportions in a 2-level hierarchical design. An intraclass correlation coefficient of 0.025 is based on ED patients in the New York Sepsis Clinical Database

Compliant ED patients with the bundle	Pre-intervention mortality, %	Intervention period	Post-intervention mortality, %	Difference	Difference in difference
3-h arm	19.1		18.9	0.2%	2.7%
1-h arm	19.1		16.2	2.9%	

Using the power estimate methods of Donner and Klar, nine hospitals per arm with a minimum of 105 patients in each hospital will give 80% power to detect a 2.7% difference-in-difference in mortality, with *α* = 0.05, ICC = 0.025, and an equal number of hospitals per arm. To account for limitations in preliminary data, we increased the target number of patients by 50% to 158 patients. Since we expect bundle compliance in the 1-h arm to be 70%, 230 *total subjects* per hospital are needed. Table 4 shows the expected overall hospital mortality in the intent-to-treat analysis based on these assumptions. Multiple imputation will be used to handle missing data.

**Table 4:** Estimated pre- and post-intervention overall mortality used in the intent-to-treat analysis

ED patients	Pre-intervention^1^	Compliant group		Non-compliant group		Post-intervention overall mortality, %
		Adherent, %	Mortality, %	Non-adherent, %	Mortality, %	
3-h arm	23.2	80	18.9	20	26.1	20.3
1-h arm	23.2	70	16.2	30	24.4	18.7

^1^Based on analysis of the New York Sepsis Database

### Analysis plan

#### Aim 1: 1-h bundle will have significantly lower patient mortality and improve all secondary outcomes

##### Primary outcome analysis

An intention to treat analysis of patient mortality in each arm will be evaluated in 2 ways: (1) the difference, in primary outcomes (hospital mortality), between the two arms during the 6-month baseline assessment and the 6-month outcomes assessment, and (2) the comparison between the first two differences (i.e., difference-in-difference estimation). This difference-in-difference analysis will be the *primary analysis* to assess the *primary hypothesis* of Aim 1: the 1-h bundle will result in lower mortality than the 3-h bundle. Both absolute and risk-adjusted hospital mortality will be reported. Random-effects logistic regression will evaluate all the mortality differences. The random term will be the hospital. This model will contain three binary risk factors: (1) 1-h arm vs. 3-h arm, (2) 6-month baseline period prior to randomization vs. 6-month outcome assessment phase, and (3) the interaction of these two terms. The effect of the intervention and the effect of the two time periods will be quantified by odds ratios while the differences in hospital mortality will be quantified by model probabilities. Additionally, the model will be adjusted for hospital level 3-h bundle compliance prior to intervention along with patient and hospital characteristics.

##### Compliance definition

This study will collect the date and time associated with each of these four measures: (1) when serum lactate level was collected, (2) when blood culture was collected, (3) when antibiotics were started, and (4) when fluids (30 ml/kg crystalloids) were started. Compliance is defined as to whether or not these date and time stamps were within 1 or 3 h (inclusive) of time zero depending on hospital randomization.

##### Subgroup analyses

A second sensitivity analysis will follow the statistical plan for the primary intention to treat analysis but use triage time as the time of presentation (“time zero”), and a third sensitivity analysis will also follow the intention to treat analysis strategy but count discharge to hospice as a death. A fourth sensitivity analysis will examine only patients who received therapies after the time of presentation to more properly evaluate the impact of therapies initiated in the hospital (e.g., excluding patients assigned credit for fluids or antibiotics administered prior to arrival to the hospital). A fifth subgroup analysis will examine the impact of time to treatment on mortality in both study arms using time as a continuous variable.

##### Secondary outcomes

Four secondary outcomes will be investigated: respiratory failure requiring mechanical ventilation, the number of ventilator-free days, and hospital and ICU length of stay. The same statistical approach described in the primary analysis will be used here; however, the analysis for the count of ventilator-free days will be based on random-effects negative binomial regression or a zero inflated negative binomial regression, depending on the distribution of the count of ventilator-free days. Similar to hospital mortality, the requirement for mechanical ventilation will be based on random-effects logistic regression. Hospital length of stay analysis will be based on a random-effects linear regression. However, prior to the analysis, length of stay will be normalized using the natural logarithm since this variable is not normally distributed. After running the random-effects linear regression, the results will be back transformed to the original units producing the ratio of the geometric mean length of stay for patients in one arm compared to the other arm.

#### Aim 2: Quantitative analysis will demonstrate improved compliance in both arms through the implementation strategy and qualitative analyses will provide insights into implementation strategy effectiveness

Surveys assessing implementation-related constructs will be completed at baseline (Exploration phase) and then repeated during the Sustainment phase. We will conduct within-subjects Wilcoxon signed rank test with the survey data, examining changes over time.

##### Quantitative analysis of bundle compliance

Bundle compliance will be defined as all-or-nothing for the individual elements of the bundles. The statistical analysis will be similar to the primary analysis for hospital mortality.

##### Qualitative analyses of bundle compliance

We will supplement quantitative analyses with qualitative interviews with key informants (ED leaders, physicians, and nurses) during the Exploration and Sustainment Phases and qualitative process evaluation data from the Implementation Phase learning collaborative meetings. For the Exploration and Sustainment Phases, we will recruit participants until thematic saturation is reached [69], anticipating up to 5 participants per site during each Phase (*N* = 90). For the Implementation Phase, learning collaborative meetings will be recorded and coded as process evaluations by the study team. Learning collaborative meetings will be recorded but will not be transcribed; instead, detailed notes will be taken during interviews and collaborative meetings. Qualitative interviews will be coded using a rapid analytic approach described by Hamilton [70], in which a template is developed to summarize transcripts. The deductive domains for this summary template will be based on the interview guides with additional space for other observations and reflections. The qualitative team will also be able to add inductive domains to the template in response to the data collected [71]. The data entered into the summary template will be analyzed using matrix analysis [72].

#### Aim 3: Identify four discrete, previously validated, sepsis phenotypes, two of which may identify patients who are significantly more likely to benefit from the 1-h bundle (γ and ∂)

Recently, the Sepsis ENdotyping in Emergency CAre (SENECA) project derived and evaluated sepsis phenotypes using clinical data from presentation in the ED. Using a variety of unsupervised clustering methods, 4 sepsis phenotypes (*α*, *ß*, *y*, and ∂) were derived, using 29 clinical variables such as vital signs, laboratory values, and demographics [73]. These phenotypes were validated in 6 studies across > 60,000 patients and found to have differences in clinical features, host response biomarkers, and clinical outcomes. In the present study, routine clinical variables will be extracted through EHR data or manual entry within the first 24 h of sepsis onset. Sepsis phenotypes will be determined using multivariate modeling. To understand if treatment effectiveness of the 1-h bundle are differentiated by phenotype, we will use unadjusted random-effects logistic regression with the following risk factors: phenotype, intervention arm, and the interaction of these two variables.

## Discussion

This study represents a critical step in assessing sepsis bundle effectiveness and implementation strategies to support compliance. It will be the first rigorous cluster-randomized design used to evaluate sepsis bundles and will compare two different bundle timelines for accomplishing early metrics. In addition, a tailored implementation strategy to enhance the implementation of sepsis bundles represents a novel approach. Rigorous formative, process, and summative evaluations using a concurrent mixed methods approach will provide important information to sites to correct site-level implementation strategy problems. In addition to our pragmatic and hybrid effectiveness-implementation aims, we will also explore heterogeneity of treatment effects in recently described clinical phenotypes. These phenotypes may respond differently to the interventions, informing future studies of bundle effectiveness.

### Design considerations

Several key design considerations for this study warrant discussion. First, we chose to compare the 1-h to the 3-h bundle. We could have proposed separate trials for 1- and 3-h bundles, comparing each to “usual care.” Because most United States hospitals now have sepsis protocols and are mandated to report compliance with the 3-h bundle [15–35], the 3-h bundle is a de facto proxy for usual care. In addition, because hospitals are mandated to report compliance with the 3-h bundle and because nationally compliance is moderate at 60% [42], the implementation strategy will be applied to both arms. We hypothesize that this strategy will increase compliance in both arms. Increased compliance should in turn assist sites in their efforts to comply with SEP-1.

Second, it is possible that hospitals in the 3-h arm may adopt features of the 1-h bundle, shortening the time to bundle completion and diluting the difference between the two study arms. We believe this is unlikely to be the case as the implementation strategy is designed to improve compliance with the 3-h bundle, not specifically to reduce time to treatment. In addition, it is unlikely that hospitals in the 3-h arm will develop strategies to approach the time requirements of the 1-h bundle. If 3-h hospitals do adopt implementation strategies that would target the 1-h bundle, Aim 2 analyses will allow assessment of this possibility.

Finally, by conducting the study among a diverse sample of hospitals from the Discovery Network and SCCM, we have considered issues related to generalizability. We have identified 18 hospitals and verified that none are outliers in terms of baseline compliance with the 3-h bundle. Conducting the study at hospitals of varying size and in both academic and community settings will enhance generalizability of the results.

### Anticipated findings and impact

We hypothesize that the 1-h bundle will be more effective than the 3-h bundle for reducing hospital mortality, respiratory failure, increasing ventilator-free days, and hospital (and ICU) length of stay. We additionally anticipate that use of the implementation strategy will improve compliance with both bundles. If these hypotheses are correct, improvement efforts should shift to adopt the 1-h bundle employing a rigorous implementation strategy. If there are no differences between the bundles, efforts should return to enhancing compliance with the 3-h bundle. A negative finding would be particularly important, since some hospitals are already focusing on the 1-h bundle in the ED. In addition, identification of sepsis phenotypes using routinely available clinical data may identify a specific groups of patients that benefit from an appropriately timed bundle.

## Trial status

Baseline data collection for the trial began December 1, 2022. Sites will be randomized to the 1- or 3-h bundle at the end of June 2023. The trial is funded through June 2027.

### Supplementary Information

**Additional file 1.**

## Data Availability

The study’s Publication Subcommittee policy stipulates that investigators who contributed data to this study will have first priority for a period of 12 months after publication of primary manuscript for ancillary studies that require the analysis and use of data collected as part of the study. After that time, de-identified study data will be available in a data repository, consistent with NIH policy.

## Abbreviations

CMS: Centers for Medicare and Medicaid Services
DSMB: Data and Safety Monitoring Board
SEP-1: Early Management Bundle: Severe Sepsis/Septic Shock
EHR: Electronic health record
ED: Emergency departments
EBPAS-15: Evidence-Based Practices Attitudes Scale
ERIC: Expert Recommendations for Implementing Change
EPIS: Exploration, Preparation, Implementation, Sustainment Framework
ICS: Implementation Climate Scale
ILS: Implementation Leadership Scale
ICU: Intensive care unit
ORIC: Organizational Readiness for Implementing Change
PRECIS: Pragmatic Explanatory Continuum Indicator Summary
SENECA: Sepsis ENdotyping in Emergency CAre
SIRS: Systematic Inflammatory Response Syndrome
SCCM: Society of Critical Care Medicine
SPIRIT: Standard Protocol Items: Recommendations for Interventional Trials
SSC: Surviving Sepsis Campaign
